# Age and Sex-Specific Relationships between Phthalate Exposures and Obesity in Chinese Children at Puberty

**DOI:** 10.1371/journal.pone.0104852

**Published:** 2014-08-14

**Authors:** Yunhui Zhang, Xiangzhou Meng, Li Chen, Dan Li, Lifang Zhao, Yan Zhao, Luxi Li, Huijing Shi

**Affiliations:** 1 Department of Environment Health, School of Public Health/Key Laboratory of Public Health Safety of Chinese Ministry of Education, Fudan University, Shanghai, China; 2 State Key Laboratory of Pollution Control and Resources Reuse, College of Environmental Science and Engineering, Tongji University, Shanghai, China; 3 Department of Maternal, Child and Adolescent Health, School of Public Health/Key Laboratory of Public Health Safety of Chinese Ministry of Education, Fudan University, Shanghai, China; INIA, Spain

## Abstract

**Objective:**

To examine the age and sex-specific associations of urine levels of six mono-phthalates with body size and fat distribution in Chinese children at puberty.

**Materials and Methods:**

Four hundred and ninety-three school-aged children (247 boys, 246 girls) were recruited. Obesity related anthropometric indices were measured and body fat proportion (BF%) was calculated. Spot urine samples were collected and phthalate monoesters were detected by an API 2000 electrospray triple quadrupole mass spectrometer (ESI-MS/MS). Associations between phthalate exposure and overweight/obesity measures and their trends were examined by multiple linear regression and Logistic regression analyses, respectively.

**Results:**

Di-2-ethylhexyl phthalate (DEHP) metabolites and monobutyl phthalate (MBP) were found to be the most detectable chemicals. In 8–10 years (yrs) group, concentrations of MEHP and MBP were significantly higher in girls than those in boys. However, concentrations of all phthalate monoesters, except for MEP and MEHP, in 11–13 yrs boys were significantly higher than those in girls. After adjusting for confounders including puberty onset, urinary concentrations of MBP and sum of low molecular-weight phthalate metabolites (∑LMP) were positively associated with boys' obesity in a concentration-effect manner, while concentrations of MEHP, MEHHP and sum of DEHP metabolites (∑MEHP) were negatively associated with girls' obesity. Associations between phthalate exposure levels and BMI z-score changes were age- and sex-specific in school-age children.

**Conclusion:**

There are age and sex-specific concentration-effect associations between phthalate exposure and fat distribution in Chinese children. Urinary phthalate levels in 11–13 yrs boys were about 30 percent higher than those in girls, and ∑MEHP levels in younger boys (<10 yrs) were significantly higher than those in elder boys (>10 yrs). Associations were positive for MBP and ∑LMP with both BMI z-score and fat distribution in boys >10 years of age, and negative for ∑MEHP with fat distribution in girls <10 years of age.

## Introduction

The rising prevalence of obesity has been of great public health concern in most countries in the world. The World Health Organization (WHO) reported that, in 2005, more than 1 billion people were overweight (body mass index (BMI) ≥25) and more than 300 million were obese (BMI ≥30) [1]. The worldwide prevalence of childhood overweight and obesity had attained 6.7% by 2010 [2]. In China, the number of obese children increased four times during 1998–2005, and the prevalence of obesity were 8.8% in girls and 15.1% in boys in Shanghai [3]–[4].

Obesity is closely linked to many adverse health effects, including type II diabetes, coronary heart disease, ischemic stroke, certain cancers (breast, colon and prostate cancer, etc.) and ultimately, mortality [5]. Globally, 44% of diabetes burden, 23% of ischemic heart disease burden and 7–14% of certain cancer burden are attributable to overweight and obesity [1]. Besides changes in diet and increasing trends of physical inactivity, exposure to environmental pollutants during the critical window period (e.g., perinatal and peripubertal periods) might be one of the risk factors linked to childhood obesity.

As one kind of environment endocrine disruptors, phthalates are commonly used as plasticizers in household products, personal care products and medical devices. The extensive human exposure was demonstrated in numerous studies by quantifying phthalate metabolites in urine samples from both adults and children. Being the PPAR-agonist, phthalate monoesters might affect lipid metabolism and adipocytes in experimental settings, which indicated that phthalate exposures might induce body weight increases in humans [6]. In two cross-sectional studies of the National Health and Nutrition Examination Survey (NHANES), researchers reported that the urinary phthalate metabolites (monoethyl phthalate (MEP) and monobutyl phthalate (MBP)) were associated with higher body mass index (BMI), waist circumference, or homeostatic model assessment (a measure of insulin resistance) in adults. These results suggested that phthalates might be the potential environmental risks of obesity in adults [7]–[8].

Several studies showed significant but not consistent associations between phthalate exposures and body sizes in children. For example, both mono-2-ethylhexyl phthalate (MEHP) and MEP concentrations were positively associated with BMI or waist circumference in Chinese children after adjustment for age, sex and other phthalate metabolites in a cross-sectional study [9]. However, data from a New York cohort study showed that only sum concentrations of low molecular-weight phthalates (LMP) were associated with BMI and waist circumference in overweight girls aged 6–8 yrs [10]. Moreover, inverse associations between urinary concentrations of phthalate metabolites and height, weight, body surface and height gain were found in Danish children [11]. Thus, there are not consistent evidence to conclude that phthalate exposure is associated with overweigh or obesity in children.

The inconsistent results might be due to inappropriate standards of overweight and obesity. In children, change in BMI percentile may not accurately reflect change in adiposity over time, especially among adolescent boys and children with lower BMI. The increase in abdominal adipose tissue and percentage of body fat (BF%), which is more strongly associated with cardiovascular disease risk than BMI is [12]–[13], may be better predictors of adverse health outcomes than BMI.

In the current cross-sectional study, we aimed to assess the exposure levels of phthalate metabolites in boys and girls during pre- and early pubertal period, investigate the associations of urine levels of six mono-phthalates with body size and fat distribution in adolescents by using various parameters, and explore the age and sex-specific differences in these associations.

## Materials and Methods

### Subject Recruitment

From October to November 2011, in the national Puberty Timing and Health Effects in Chinese Children (PTHEC) study, a total of 2,007 school students (Grade 1 to 12) were recruited from a suburban district in Shanghai by a stratified multi-stage cluster sampling method to collect anthropometric measurements, pubertal development status and related factors. Of these subjects, 503 students of grade 3 to grade 7 were randomly selected for further laboratory analysis of urine samples. A set of questionnaires were completed by the students and their guardian that included questions on perinatal factors, socio-demographic variables, physical activity, dietary habit and intake, and spermarche in boys or menarche in girls. The study was approved by the Institutional Review Board (IRB) of Fudan University. Parents of all students received a consent form stating the study purpose, process and voluntary nature of participation. Parents were asked to inform teachers if they did not want their children participate in the study. All students were also informed by their teachers about the study purpose, the processes involved, urine samples collection, and voluntary nature of participation, and were reminded again at the time of data collection. The ethics committees/IRBs approved the opt out consent procedure for the students.

### Urinary Sample Collection and Measurement

Morning spot urine samples were obtained from children on the day when administering questionnaires. All specimens were collected with glass vessels to avoid contamination of phthalates during handling and storage. Frozen samples were stored in phthalate-free containers and transferred with dry ice to the Fudan University laboratory for analysis.

Analyses of six phthalate monoesters (MEP, MBP, monomethyl phthalate [MMP], mono-2-ethylhexyl phthalate [MEHP], mono(2-ethyl-5-oxohexyl) phthalate [MEOHP] and mono(2-ethyl-5-hydroxyhexyl) phthalate [MEHHP]) in urinary samples were described elsewhere [14]–[15]. In brief, the determination of phthalate metabolites in urinary (0.5 mL) involved enzymatic deconjugation of the glucuronidated metabolites, solid-phase extraction, separation with an Agilent 1100 Series high-performance liquid chromatography (HPLC) system (Agilent Technologies, Santa Clara, CA), and detection by an API 2000 electrospray triple quadrupole mass spectrometer (ESI-MS/MS; Applied Biosystems, Foster City, CA). ESI-MS/MS analysis was performed at Fudan University's Key Laboratory of Ministry of Education on Public Health Safety, using the manufacturer's reagents and following the manufacturer's protocol. Chromatographic separation was achieved using a Waters ACQUITY UPLC HSS T3 column (100 mm×2.1 mm, 1.8 µm). Target compounds were determined by multiple-reaction monitoring (MRM) in the negative ionization mode. For instrumental analysis, two isotopically labeled phthalate metabolites (^13^C_4_-MBP and ^13^C_4_-MEHHP) and ^13^C_4_-4-methylumbelliferone were used as internal standards. The limit of detection (LOD) was 0.25 to 0.5 ng/mL. Lab analysts were blind to all information concerning our subjects.

Phthalate metabolites were adjusted by specific gravity to correct for urinary dilution, as recommended by Hauser et al [16]. Specific gravity was measured using a handheld refractometer (Atago PAL 10-S, Tokyo, Japan). The correction formula was P_c_ = P×[(1.024–1)/(SG–1)], where Pc is the specific gravity- corrected phthalate metabolite concentration, P is the experimental phthalate metabolite concentration, and SG is the specific gravity of the urine sample [9].

### Anthropometric measurements

Obesity related anthropometric indices, including body weight, height, waist circumference, hip circumference, tricep and subscapular skinfold thicknesses, were measured in all children according to methods and standardization protocols for measurement recommended by WHO [17]. Calibrated instruments (e.g., scales and meters for measuring height) were used. The indices were measured twice by two trained technicians after urine samples were collected. The allowed differences between the measurements of two observers were set at 0.1 kg for weight, 0.7 cm for length, 0.5 cm for circumferences, and 0.1 cm for skinfolds at maximum. The final value used for assessing obesity was the average of each pair of measurements.

BMI was calculated as weight (in kilogram) divided by square of height (in square meter), and established method was used to compute BMI z-score [18]. Body fat proportion (BF%) was calculated with the Yao's formula, which was widely used for Chinese school age children from 7–12 years [19]. The formula were BF%  = 6.931+0.428X for boys and BF% = 7.896+0.458X for girls, respectively; X (in mm) was skin fold thickness (triceps+ subscapular). BSA was calculated with the Haycock formula, which was BSA(m^2^) = 0.007184×height(cm)^0.725^×weight(kg)^0.425^ [20].

Breast stages (B1-B5) and pubic hair stages (PH1-PH5) were assessed by inspection and palpation according to the methods by Marshall & Tanner [21]–[22]. Testicular volume (TV) was estimated by palpation to the nearest 1 mL using Prader's orchidometer and divided into four levels (T1–T4) as <4 mL, 4≤TV<12 mL, 12≤TV<20 mL, and ≥20 mL [23]–[24]. In case of discrepancy between left and right side, the largest measurement was used for classification. All assessments were performed privately by a female pediatrician (for girls) or a male urologist (for boys). Pubertal onset was defined as the first appearance of testicular volume ≥4 mL (T2) in boys and the first appearance of breast buds (B2) in girls.

### Data Analysis

Parametric t-tests and χ^2^ tests were used to compare demographic characteristics in boys and girls. Phthalate concentrations were skewed to the right, and medians and geometric mean (GM) were used to describe phthalate concentrations. Log transformation was applied to phthalate concentrations, waist-hip ratio (WHR), subscapular skinfold thickness, triceps skinfold thickness, and BF% to improve the approximation of normal distribution. For phthalate metabolite levels below the LOD, the value of the LOD divided by the square root of 2 was used. We calculated the sum of DEHP metabolites (MEHP, MEHHP, and MEOHP) as ΣMEHP and combined low molecular-weight phthalate metabolites (MBP, MMP and MEP) as ∑LMP and all detectable monoesters as ∑All. Spearman correlation was used to examine the relationships among individual phthalate concentrations.

Multiple linear regression analyses were performed with the log-transformed data (WHR, subscapular skinfold thickness, triceps skinfold thickness, and BF%) and normally distributed data (BMI, BMI z-score, waist circumference, hip circumference, and body surface area (BSA)) as outcome variables. Categorical analyses by exposure quartiles were also performed. The distribution of each phthalate was divided into quartiles except for MEP. For low concentrations of MEP (almost 50 percent of MEP concentrations below the LOD), we defined the levels below the LOD as quartile 1, and the other data were separated into three equal sections.

Phthalate concentrations were treated as both continuous variables and categorical variables in the Logistic regression analyses. As continuous variable, relationship between phthalate concentration and overweight or obesity was tested with p-value for trend. Trends in ORs of overweight/obesity measures across increasing phthalate categories were determined by modeling phthalates as ordinal variables. By taking the lowest quartile as the reference, the odds ratio (OR), 95% confidence interval (CI), and p values of overweight/obesity for phthalates were estimated for each quartile after adjusting for socio-economic level, physical activity, dietary nutriment intake and puberty onset. Because phthalate metabolite concentrations were correlated with each other, urine phthalate metabolites were also included as covariates in the models. The analyses were considered statistically significant when P <0.05. All statistical analyses were conducted using SAS (version 9.1, SAS Institute Inc.).

## Results

Given that BMI is not a reliable measure of fat mass in children as in adults, we used weight as the primary measure of overweight/obesity [25]. We used the 80^th^ and 90^th^ percentiles of age- and gender-specific weight distribution in population as cutoffs for overweight and obesity based on the published survey [26]. Meanwhile, we excluded 10 marasmus children whose weight for age was lower than 10^th^ percentiles of gender-specific weight distribution. According to aforementioned criteria, a total of 493 school students (247 boys, 246 girls) randomly selected from the PTHEC attended this study. Of those, 55 boys and 35 girls were identified as overweight, 65 boys and 23 girls were considered as obesity, and 127 boys and 188 girls were defined as normal weight.

For participants at puberty in this study, children's height, weight and pubertal onset rate increased while their WHR decreased with age (Table 1). In 8–10 yrs and 11–13 yrs groups, the puberty onset rates were 10.1% and 82.6% in boys and 55.9% and 96.3% in girls, respectively. After adjustment for age, boys had significant larger values of body weight, BSA, BMI and indicators of body fat distribution (waist circumference, hip circumference, WHR, subscapular skinfold thickness, triceps skinfold thickness and BF%) compared with girls.

**Table 1:** Anthropometric characteristics of 493 children at puberty.

Characteristics	Boys (N = 247)		Girls (N = 246)	
	8–10 yrs	11–13 yrs	8–10 yrs	11–13 yrs
Height (cm)	137.63±7.07	152.76±9.96	136.99±7.58	151.71±8.46
Weight (kg)	37.31±9.77	49.20±15.32	32.37±7.65	44.84±11.26
BSA (m^2^)	1.18±0.17	1.43±0.24	1.11±0.14	1.37±0.18
BMI (kg/m^2^)	19.42±3.70	20.69±4.68	17.09±2.93	19.30±3.92
Waist circumference (cm)	64.67±10.16	70.01±12.44	58.60±8.32	65.73±9.55
Hip circumference (cm)	76.26±8.39	85.13±10.64	70.55±7.09	82.12±9.04
WHR^a^	0.84(0.83,0.85)	0.82(0.81,0.83)	0.83(0.82,0.84)	0.80(0.79,0.81)
Subscapular skinfold thickness^a^ (cm)	12.65(11.34,14.12)	12.84(11.62,14.19)	8.49(7.81,9.23)	10.94(10.13,11.82)
Triceps skinfold thickness^a^ (cm)	15.62(14.41,16.92)	15.42(14.26,16.67)	12.03(11.18,12.95)	13.70(12.83,14.62)
BF%^a^	0.24(0.22,0.27)	0.23(0.22,0.25)	0.14(0.13,0.15)	0.18(0.17,0.19)
Pubertal rate	10.09%	82.61%	55.86%	96.3%

a GM(95%CI).

In the present study, sources of phthalate exposures are likely to be steady because the patterns of use of personal care products, diet and other daily activities are relatively constant (data not shown). A positively skewed distribution was found for phthalate monoester levels in children. Except for MEP, more than 80% of the urinary samples had quantifiable levels of phthalate monoesters, with a higher detection rate in boys compared with that in girls (Table 2). There were age and sex-specific difference in phthalate monoester concentrations (median and GM). In 8–10 yrs groups, concentrations of MEHP and MBP were significantly higher in girls than those in boys. However, concentrations of MBP, MMP, ∑LMP, ∑MEHP metabolites and ∑All in 11–13 yrs boys were significantly higher than those in girls. Compared with the elder boys, the younger boys had higher levels of urinary phthalate metabolites except for MBP. Furthermore, up to 2-fold higher levels of MBP, MMP, and di-2-ethylhexyl phthalate (DEHP) metabolites, including MEHP, MEHHP, MEOHP and ∑MEHP, were observed in younger girls (Table 2).

**Table 2:** Age and sex-specific phthalate metabolite concentrations (µg/L) in urine samples of school children.

Age	Phthalates (µg/L)	Boys (N = 247)					Girls (N = 246)				
		DF^a^ (%)	Median	GM	Min	Max	DF^a^ (%)	Median	GM	Min	Max
8–10 yrs	MBP	100	30.4	26.8	ND	164.9	99	35.6^b^	38.3^c^	8.4	164.9
	MMP	100	16.1^b^	16.3	3.4	245.8	100	16.8^b^	16.3	3.6	122.6
	MEP	51	ND^b^	0.8	ND	126.2	61	1.0	1.1	ND	51.7
	ΣLMP	100	37.4	37.6	6.9	282.7	100	36.7^b^	36.7	11.4	232.7
	MEHP	88	3.0^b^	2.2	ND	28.7	97	3.3^b^	3.5^c^	ND	92.2
	MEHHP	100	18.1^b^	19.4	4.7	250.7	100	20.7^b^	21.5	3.2	290.0
	MEOHP	100	6.6^b^	6.8	1.6	96.0	96	6.3^b^	6.8	1.2	115.5
	ΣMEHP	100	27.2^b^	29.6	6.7	375.4	100	32.0^b^	32.5	5.2	497.7
	ΣAll	100	62.9	62.0	11.7	455.6	100	56.1^b^	57.6	13.5	593.2
11–13 yrs	MBP	100	29.5	29.8^c^	3.6	126.4	93	20.9	13.1	ND	133.5
	MMP	100	14.3	13.0^c^	1.0	101.3	99	9.9	9.3	ND	126.0
	MEP	69	1.2	1.4	ND	84.1	54	ND	1.1	ND	422.0
	ΣLMP	100	42.5	42.3^c^	10.2	146.3	100	31.5	25.5	0.6	497
	MEHP	85	1.6	1.4	ND	40.9	83	1.1	1.1	ND	117.1
	MEHHP	100	15.0	14.4^c^	1.5	158.3	99	11.3	11.5	0.8	508.4
	MEOHP	99	5.7	5.2^c^	0.6	54.3	96	3.9	3.8	ND	238.8
	ΣMEHP	100	22.7	21.9^c^	2.4	249.7	100	16.5	16.8	1.3	864.4
	ΣAll	100	59.7	61.7^c^	13.1	295.0	100	48.3	40.8	2.0	978.2

a Detection frequency.

b Analysis of variance adjusted for sex, p<0.05.

c Analysis of variance adjusted for age, p<0.05.

DEHP metabolites and MBP were the most detectable chemicals in current study. The possible reason was that their diesters were the most frequent products of plasticizers in China. Spearman correlation analyses showed that MEHP level was significantly associated with its secondary metabolites (MEHHP and MEOHP) and MBP level in urine (data not shown). It meant that other metabolites should be included as confounders in models for evaluating the association between one metabolite and body fat distribution and obesity.

**Figure 1:**
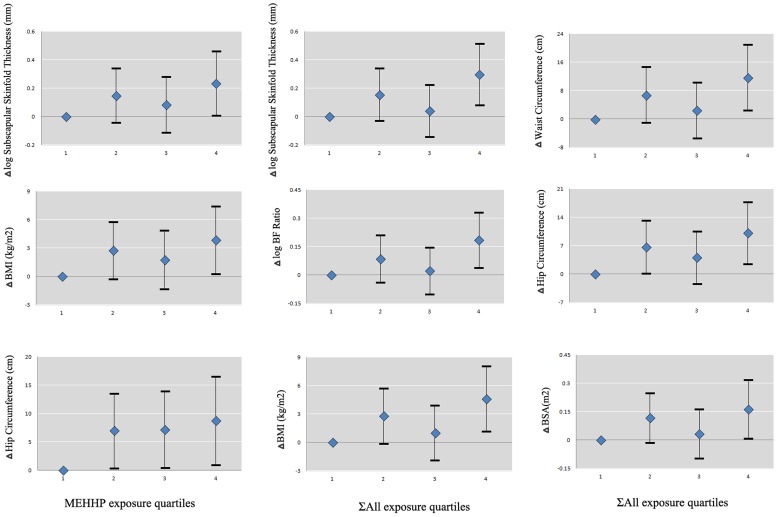
Associations between exposure quartiles for log-transformed concentrations of MEHHP and ∑All monoesters and body fat distribution indices with significant continuous regression coefficients in 8-10yrs boys of China, corrected for puberty onset, socio-economic level, physical activity and dietary nutriment intake. (Blue rhombus: regression coefficient; Error bar: 95% confidence interval; log-transformed indices including subscapular skinfold thickness, triceps skinfold thickness and BF ratio; BMI, BMI z-score, waist circumference, hip circumference, and BSA were normally distributed data)

With the increase of MEHHP and ∑All, body fat indexes and BSA were decreased in 8–10 years boys. The highest quartile of ∑All was significantly associated with subscapular skinfold thickness, waist circumference, hip circumference, BF%, BMI and BSA, compared with the lowest quartile. In coincidence with this, the highest quartile of MEHHP level was associated with subscapular skinfold thickness and BMI, while other quartiles were positively associated with hip circumference (Figure 1).

**Figure 2:**
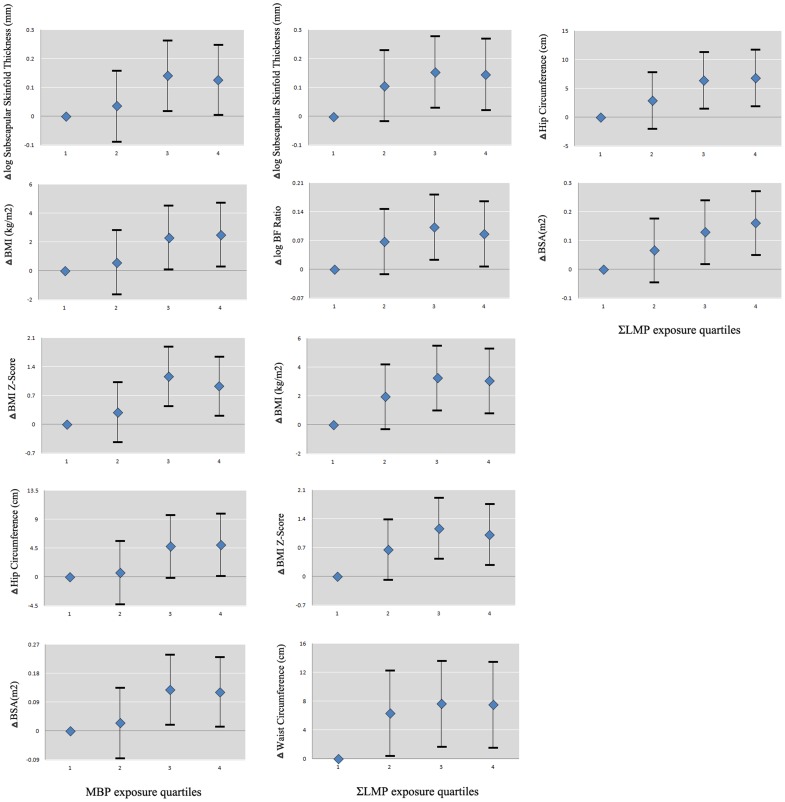
Associations between exposure quartiles for log-transformed concentrations of MBP and ∑LMP and body fat distribution indices with significant continuous regression coefficients in 11-13yrs boys of China, corrected for puberty onset, socio-economic level, physical activity and dietary nutriment intake. (Blue rhombus: regression coefficient; Error bar: 95% confidence interval; log-transformed indices including subscapular skinfold thickness, triceps skinfold thickness and BF ratio; BMI, BMI z-score, waist circumference, hip circumference, and BSA were normally distributed data)

In the 11–13 yrs group, ∑LMP level was positively associated with all measured obesity indices including subscapular skinfold thickness, waist and hip circumferences, BF%, BMI, BMI z-score and BSA. Noticeably, MBP was the main contributive phthalate that was associated with the growth of boys. With the increased level of MBP in urine, the correlation coefficients with body size and fat distribution indices were increased. The third and forth quartiles of MBP were significantly associated with higher BSA, BMI, BMI z-score, subscapular skinfold thickness and hip circumference (Figure 2).

After adjusting for the aforementioned confounders, MBP and ∑LMP concentrations in the highest quartile were associated with boys' obesity in a concentration-effect manner. In girls, negatively significant associations were found between urinary MEHP, MEHHP and ∑MEHP levels and obesity (p-value for trend<0.05). No difference between overweight children and controls was observed for all detectable phthalates (Table 3).

**Table 3:** Associations between log-transformed urinary phthalate metabolite concentrations and overweight/obesity in 493 school children of China.

Gender	Phthalate (µg/L)	Quartiles	Overweight vs Normal			Obesity vs Normal		
			OR	95% CI	p value	OR	95% CI	p value
Boys (OB∶OV∶NO = 65∶55∶127)^a^	MBP	Q1	1	Ref	/	1	Ref	/
		Q2	0.951	(0.312,2.893)	0.929	2.115	(0.705,6.346)	0.181
		Q3	1.885	(0.636,5.582)	0.253	2.494	(0.832,7.475)	0.103
		Q4	1.156	(0.339,3.947)	0.817	5.768	(1.622,20.515)	0.007^c^
		p-trend	0.413			0.032^b^		
	ΣLMP	Q1	1	Ref	/	1	Ref	/
		Q2	0.945	(0.316,2.826)	0.919	3.945	(1.305,11.927)	0.015^c^
		Q3	1.148	(0.417,3.161)	0.790	3.994	(1.291,12.351)	0.016^c^
		Q4	1.299	(0.443,3.809)	0.633	6.841	(2.073,22.575)	0.002^c^
		p-trend	0.668			0.037^b^		
	ΣAll	Q1	1	Ref	/	1	Ref	/
		Q2	1.100	(0.386,3.137)	0.858	2.790	(1.034,7.534)	0.043^c^
		Q3	1.007	(0.362,2.802)	0.990	3.375	(1.138,10.012)	0.028^c^
		Q4	1.240	(0.425,3.617)	0.693	3.299	(1.104,9.856)	0.033^c^
		p-trend	0.852			0.331		
Girls (OB∶OV∶NO = 23∶35∶188)^a^	MEHP	Q1	1	Ref	/	1	Ref	/
		Q2	0.743	(0.295,1.869)	0.528	0.998	(0.287,3.468)	0.998
		Q3	1.071	(0.415,2.763)	0.888	0.246	(0.043,1.410)	0.115
		Q4	0.664	(0.230,1.913)	0.448	0.128	(0.013,1.242)	0.076
		p-trend	0.433			0.024^b^		
	MEHHP	Q1	1	Ref	/	1	Ref	/
		Q2	1.256	(0.450,3.507)	0.664	0.829	(0.218,3.150)	0.783
		Q3	1.329	(0.483,3.656)	0.582	0.278	(0.056,1.389)	0.119
		Q4	1.047	(0.339,3.232)	0.937	0.084	(0.008,0.910)	0.042^c^
		p-trend	0.838			0.033^b^		
	MEOHP	Q1	1	Ref	/	1	Ref	/
		Q2	0.816	(0.291,2.284)	0.698	0.738	(0.195,2.795)	0.654
		Q3	1.090	(0.405,2.931)	0.864	0.225	(0.044,1.159)	0.075
		Q4	0.929	(0.305,2.829)	0.897	0.092	(0.009,0.958)	0.046^c^
		p-trend	0.759			0.050		
	ΣMEHP	Q1	1	Ref	/	1	Ref	/
		Q2	0.839	(0.312,2.257)	0.728	0.562	(0.152,2.080)	0.388
		Q3	0.965	(0.363,2.567)	0.943	0.237	(0.049,1.140)	0.072
		Q4	0.811	(0.273,2.405)	0.705	0.078	(0.008,0.791)	0.031^c^
		p-trend	0.746			0.029^b^		
	ΣAll	Q1	1	Ref	/	1	Ref	/
		Q2	0.655	(0.246,1.747)	0.398	0.402	(0.105,1.547)	0.185
		Q3	0.683	(0.260,1.792)	0.438	0.070	(0.008,0.633)	0.018^c^
		Q4	1.218	(0.473,3.137)	0.683	0.288	(0.063,1.309)	0.107
		p-trend	0.956			0.084		

a OB means obesity, OV means overweight, NO means normal weight.

b Logistic regression analysis adjusted for socio-economic level, physical activity, dietary nutriment intake and puberty onset, phthalate metabolite concentrations as continuous variables, p<0.05.

c Logistic regression analysis of variance adjusted for socio-economic level, physical activity, dietary nutriment intake and puberty onset, phthalate metabolite concentrations as ordinal variables, p<0.05.

**Figure 3:**
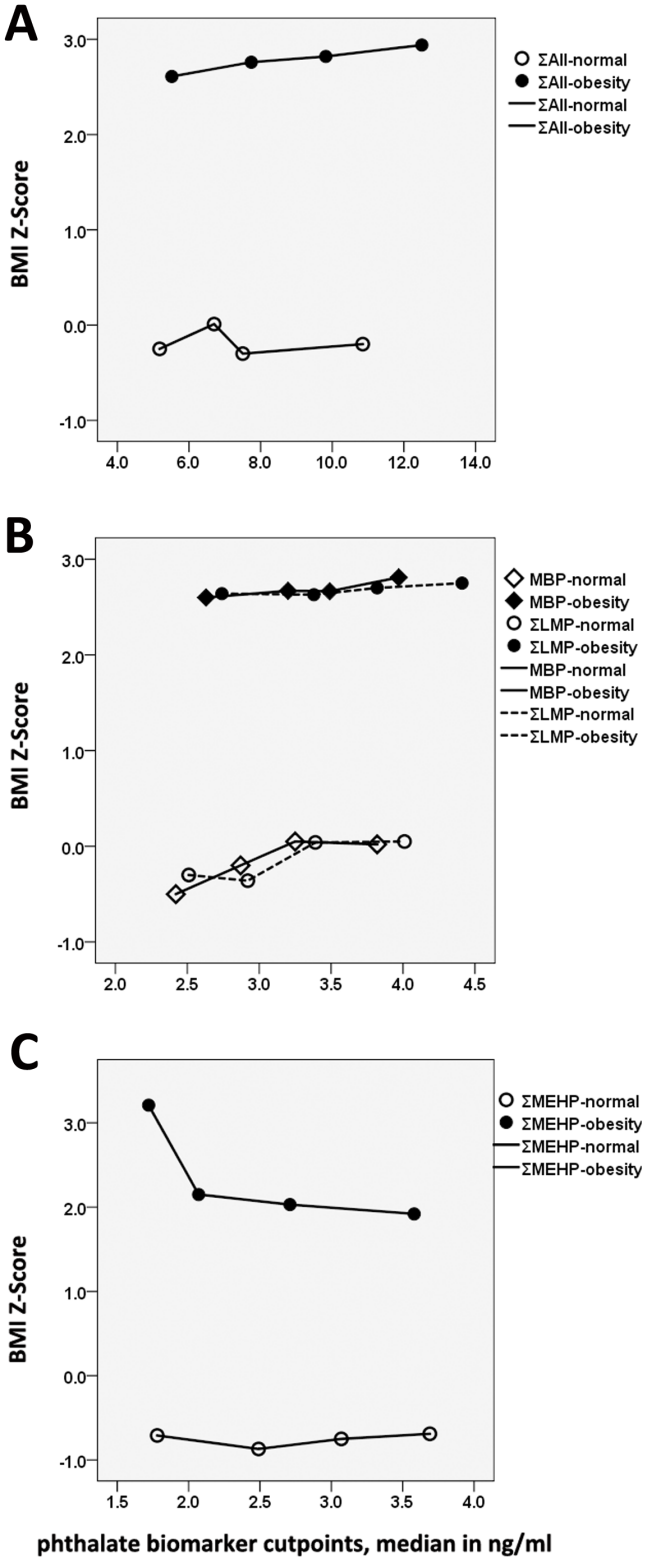
BMI z-score in relation to urinary phthalate metabolite concentrations, by median (ppb) quartiles for ∑All in 8-10yrs boys (A), MBP and ∑LMP in 11-13yrs boys (B), and ∑MEHP in 8-10yrs girls (C). Obesity and normal weight children were stratified by age- and gender-specific weight distribution based on the national survey in Chinese school children, normal weight children (<80th %) and obesity children (≥90th%), National Puberty Timing and Health Effects in Chinese Children (PTHEC) study, Shanghai 2010-2012.

Consistently, associations between phthalate exposure levels and BMI z-score are age- and sex-specific in school-age children (Figure 3). In 8–10 yrs girls, obese girls had higher BMI z-scores and lower ∑MEHP metabolite levels than girls with normal body weight. In 8–10 yrs obese girls, BMI z-score decreased with increased ∑MEHP levels (Figure 3C). In 11–13 years old boys, obese children had higher BMI z-scores and MBP/∑LMP levels than those with normal body weight. With the increasing in MBP and ∑LMP concentrations, BMI z-score increased in both obese and normal weight boys (Figure 3B). Similarly, with the increasing ∑All phthalate levels, BMI z-score increased in 8–10 yrs obese boys.

## Discussion

As one of the environmental endocrine disruptors, phthalates are ubiquitous in environment. Animal studies showed that phthalates were reproductive and developmental toxicants. In vitro systems, certain phthalate metabolite, such as MEHP, was also considered as a potential obesogen that could increase glyceroneogenesis and fatty acid reesterification in human adipocytes. Previous epidemiological data showed that monoethyl phthalate (MEP) was associated with higher BMI or waist circumference, while MEHP was related to decreased BMI in females >12 yrs [7]–[8]. Furthermore, monoisobutyl phthalate (MiBP) was related to increased fat amount in the subcutaneous abdominal region in women measured by dual-energy X-ray absorptiometry (DXA) and abdominal magnetic resonance imaging (MRI) two years later [27]. These population-based studies reported some positive associations between phthalate exposure and body weight increases, but did not provide sufficient evidence to conclude the age- and gender-specific difference in the associations of phthalate with obesity.

Usually, BMI could be used to estimate growth of prepubertal children. However, BMI z-score and BF% were more suitable than BMI to evaluate the growth of adolescent at the stage of early puberty. In our study, three kinds of measurements were used to assess the development of children at pre- and early puberty. BSA reflected the body size changes, while waist circumference, hip circumference, subscapular skinfold thickness, triceps skinfold thickness and BF% reflected the body fat distributions. Furthermore, BMI and BMI z-score were integrated indices that systematically reflect changes both in body size changes and in body fat distribution.

In the present study, we investigated the puberty onset status, growth and body weight distribution in 247 boys and 246 girls, and measured their phthalate levels in urine samples. There was no association observed between MEP and MMP levels and body fat distribution. In contrast, positive associations were observed between MBP and ∑LMP and BMI z-score and fat distribution in boys >10 years of age, and negative associations were observed between ∑MEHP and fat distribution in girls <10 years of age. These results indicated that low-molecular-weight and high-molecular-weight phthalate metabolites might have opposite associations depending on developmental stages, which is also consistent with recently published reports that indicated the association of urine phthalates with obesity in children and adolescents [9]–[11], [27]–[28].

In our subjects, with the increasing of age group, the proportion of those who had started puberty increased from 10% to 83% in boys and from 56% to 96% in girls. Meanwhile, urinary phthalate levels increased in boys but decreased in girls, and MBP and MEHP were the most detectable phthalates in these children. Results from linear regression analyses showed that MEHP and its metabolites mainly associated with abdomen fat distributions in children <10 yrs, while MBP was mostly associated with BMI z-score and BF% in boys >10 yrs. Experiments showed that phthalates might promote the differentiation of human preadipocytes to adipocytes and thus contribute to obesity. However, our results indicate that this effect has chemical and gender-specific difference. MBP is likely to be stored in abdomen lipoid in boys, while MEHP might distribute into the adipose of whole body in girls.

It has been shown that environmental risk factors are more likely to affect weight among girls than boys [29]. However, we found that urinary phthalate metabolite levels in 11–13 yrs boys were about 30 percent higher than those in girls. ∑MEHP levels in younger boys (<10 yrs) were significantly higher than those in elder boys (>10 yrs), which might explain why higher subscapular skinfold thickness and BF% were found in 8–10 yrs boys. The most noticeable finding was the significant relationship of MBP with BMI z-score and male development. Associations of concurrent MEHP exposure biomarkers with development and obesity in our girls were not strong, but what we observed were among the phthalates with the highest levels and is consistent with our prior hypotheses and literatures [6], [28]. Peroxisome proliferator-activated receptor (PPAR) plays a key role in phthalates' adverse effects in humans. PPARγ activity is similar in rodents and humans, but stronger PPARα activity in mice than in humans may mask effects mediated through PPARγ. Animals treated with relatively high doses of phthalates such as DEHP typically displayed decreased body weight and fat mass [30]. However, when the normal mouse PPARα gene was replaced with the human PPARα gene, mice treated with DEHP gained weight and had increased epididymal white adipose mass compared with wild-type animals [31]–[32]. Thus, to understand activity differences between PPARα and PPARγ is crucial for explaining gender-depending effects of phthalates on body weight [33]–[34].

The strengths of our study include concentration-effect relationship and consistent fat distribution measures, and being consistent with findings from recent human studies. Moreover, we controlled for known risk factors for childhood obesity including puberty onset, dietary factors and physical activity. Though only one spot urine sample was collected in our study, urine phthalates has been reported to be relatively stable. Because of the ubiquity of these phthalates in Chinese adolescents, a small effect size in this study may influence significant proportion of the population. However, genetic and gender differences in exposure and development, suggest that cohort studies with larger sample size are needed in the future.
